# Kaposi’s Sarcoma-Associated Herpesvirus ORF67.5 Functions as a Component of the Terminase Complex

**DOI:** 10.1128/jvi.00475-23

**Published:** 2023-06-05

**Authors:** Yuki Iwaisako, Tadashi Watanabe, Youichi Suzuki, Takashi Nakano, Masahiro Fujimuro

**Affiliations:** a Department of Cell Biology, Kyoto Pharmaceutical University, Kyoto, Japan; b Department of Virology, Graduate School of Medicine, University of the Ryukyus, Okinawa, Japan; c Department of Microbiology and Infection Control, Faculty of Medicine, Osaka Medical and Pharmaceutical University, Osaka, Japan; Lerner Research Institute, Cleveland Clinic

**Keywords:** KSHV, Kaposi’s sarcoma-associated herpesvirus, bacterial artificial chromosome, capsid formation, genome cleavage, genome replication, herpesvirus, lytic replication, terminal repeats, terminase

## Abstract

Kaposi’s sarcoma-associated herpesvirus (KSHV) is a double-stranded DNA (dsDNA) gammaherpesvirus with a poorly characterized lytic replication cycle. However, the lytic replication cycle of the alpha- and betaherpesviruses are well characterized. During lytic infection of alpha- and betaherpesviruses, the viral genome is replicated as a precursor form, which contains tandem genomes linked via terminal repeats (TRs). One genomic unit of the precursor form is packaged into a capsid and is cleaved at the TR by the terminase complex. While the alpha- and betaherpesvirus terminases are well characterized, the KSHV terminase remains poorly understood. KSHV open reading frame 7 (ORF7), ORF29, and ORF67.5 are presumed to be components of the terminase complex based on their homology to other terminase proteins. We previously reported that ORF7-deficient KSHV formed numerous immature soccer ball-like capsids and failed to cleave the TRs. ORF7 interacted with ORF29 and ORF67.5; however, ORF29 and ORF67.5 did not interact with each other. While these results suggested that ORF7 is important for KSHV terminase function and capsid formation, the function of ORF67.5 was completely unknown. Therefore, to analyze the function of ORF67.5, we constructed ORF67.5-deficient BAC16. ORF67.5-deficient KSHV failed to produce infectious virus and cleave the TRs, and numerous soccer ball-like capsids were observed in ORF67.5-deficient KSHV-harboring cells. Furthermore, ORF67.5 promoted the interaction between ORF7 and ORF29, and ORF29 increased the interaction between ORF67.5 and ORF7. Thus, our data indicated that ORF67.5 functions as a component of the KSHV terminase complex by contributing to TR cleavage, terminase complex formation, capsid formation, and virus production.

**IMPORTANCE** Although the formation and function of the alpha- and betaherpesvirus terminase complexes are well understood, the Kaposi’s sarcoma-associated herpesvirus (KSHV) terminase complex is still largely uncharacterized. This complex presumably contains KSHV open reading frame 7 (ORF7), ORF29, and ORF67.5. We were the first to report the presence of soccer ball-like capsids in ORF7-deficient KSHV-harboring lytic-induced cells. Here, we demonstrated that ORF67.5-deficient KSHV also formed soccer ball-like capsids in lytic-induced cells. Moreover, ORF67.5 was required for terminal repeat (TR) cleavage, infectious virus production, and enhancement of the interaction between ORF7 and ORF29. ORF67.5 has several highly conserved regions among its human herpesviral homologs. These regions were necessary for virus production and for the interaction of ORF67.5 with ORF7, which was supported by the artificial intelligence (AI)-predicted structure model. Importantly, our results provide the first evidence showing that ORF67.5 is essential for terminase complex formation and TR cleavage.

## INTRODUCTION

Kaposi’s sarcoma-associated herpesvirus (KSHV) is a double-stranded DNA (dsDNA) human herpesvirus that belongs to the gammaherpesvirinae subfamily (1). Other human herpesviruses including KSHV are categorized into three subfamilies: alphaherpesvirinae (herpes simplex virus 1 [HSV-1], herpes simplex virus 2 [HSV-2], and varicella-zoster virus [VZV]), betaherpesvirinae (human cytomegalovirus [HCMV], human herpesvirus 6 [HHV-6], and human herpesvirus 7 [HHV-7]), and gammaherpesvirinae (Epstein-Barr virus [EBV] and KSHV) (2). KSHV is the etiologic agent of Kaposi’s sarcoma, primary effusion lymphoma, multicentric Castleman’s disease, and KSHV-associated inflammatory cytokine syndrome (3–6). KSHV has two states of infection, latent and lytic. The expression of a viral protein referred to as the replication and transcription activator (RTA/open reading frame 50 [ORF50]) induces the transition from latent to lytic infection, which produces progeny virions (7). The KSHV genes that are expressed during lytic infection are classified as immediate early (IE), delayed early (DE), and late (L) based on differences in the timing of expression and the conditions required for expression (2). During lytic infection, viral capsids are formed in the nucleus of the host cell, and the replicated viral genome is packaged into capsids to form a mature capsid. The mechanism responsible for mature capsid formation is still largely unknown for KSHV, but it is relatively well understood for HSV-1.

HSV-1 forms procapsids, A-capsids, B-capsids, and C-capsids during mature capsid formation (8, 9). First, a porous, hollow procapsid is formed with a globular outer layer consisting of major capsid proteins and a globular inner layer consisting of scaffold proteins (10, 11). The outer layer structure of the procapsid is supported by scaffold proteins, and the outer layer and scaffold proteins are linked together (12). Subsequently, when the link between the outer layer of the procapsid and the scaffold proteins is detached by the viral protease VP24, a globular scaffold inner layer remains inside the outer layer, and the outer layer undergoes spontaneous angularization to form a regular icosahedron. This regular icosahedron capsid, harboring a globular inner layer, is referred to as the B-capsid (13–15). Since both procapsids and B-capsids harbor a globular scaffold protein layer, it is difficult to distinguish between them in images obtained by conventional transmission electron microscopy (TEM). The A-capsids are empty capsids that have lost the scaffold proteins (16, 17). The C-capsid is the mature capsid, which lacks the scaffold proteins and contains the viral genome (16, 17).

In the herpesviral lytic replication process, multiple copies of the herpesvirus genome are replicated as a tandemly repeated precursor form, and one unit of the viral genome is cleaved at the terminal repeat (TR) as it is packaged into a capsid (18). KSHV genome consists of a unique cording region (including approximately 96 ORFs) flanked by multiple TRs, and a single TR is GC-rich 801 bp DNA sequence (3). The single unit of the viral genome is packaged and cleaved by the viral terminase complex, which consists of three viral proteins. In KSHV, it has been hypothesized that ORF7, ORF29, and ORF67.5 comprise the terminase complex based on their sequence homology to other herpesviral terminase complex proteins. However, the details regarding the structural and functional properties of the KSHV terminase complex are largely unknown.

In HSV-1, UL15 (KSHV ORF29 homolog), UL28 (KSHV ORF7 homolog), and UL33 (KSHV ORF67.5 homolog) are components of the terminase complex (19–21). These HSV-1 proteins form a tripartite complex, and the hexameric ring of this tripartite complex becomes an HSV-1 terminase (21). UL15 and UL28 as well as UL28 and UL33 interact directly, but UL15 and UL33 do not interact in the absence of UL28 (22). If any of the components are defective, the viral genome is neither encapsidated nor cleaved, and capsid formation is arrested at the B-capsid stage (23–28). Since these capsid formation pathways consist of virus-specific machineries that do not exist in any human cells, they may be targeted for the development of antiviral drugs. In fact, letermovir, a drug that targets the HCMV terminase complex, has been used clinically for HCMV treatment and exhibits high antiviral activity and low adverse events (29, 30).

In contrast to HSV-1 and HCMV, little is known about the function of the KSHV terminase complex. ORF7, ORF29, and ORF67.5 are putative components of the KSHV terminase complex. We previously reported that there was no direct interaction between ORF29 and ORF67.5. However, we found that ORF7 interacted with both ORF29 and ORF67.5 (31). We also showed that ORF7-deficient KSHV formed numerous immature soccer ball-like capsids in the nucleus and failed to cleave the KSHV TR (32). The soccer ball-like capsid was a characteristic immature capsid that contains many particulate structures. Moreover, the soccer ball-like capsid was derived from procapsid (32). While these two reports indicate that ORF7 plays crucial roles in both KSHV terminase function and capsid formation, the function of ORF67.5 is completely unknown. Therefore, this study focused on the physiological function and virological significance of KSHV ORF67.5.

Other herpesviral homologs of KSHV ORF67.5 include HSV-1 UL33, HSV-2 UL33, VZV ORF25, EBV BFRF1A, HCMV UL51, HHV-6 U35, and HHV-7 U35. KSHV ORF67.5 is 80 amino acids (aa) in length and is the shortest of the ORF67.5 homologs. HSV-1 UL33 is required for the cleavage of a single unit of the viral genome and its encapsidation. Interestingly, B-capsids accumulate in UL33-deficient HSV-1-infected cells (27, 28). VZV ORF25-deficient virus is unable to produce infectious virions (33). HCMV UL51 is also required for the cleavage of a single unit of the viral genome, and knockdown of HCMV UL51 results predominantly in B-capsid formation (34). There are no reports on the functions of HHV-6 U35 and HHV-7 U35. EBV BFRF1A is required for virus production and deletion results in B-capsid (35).

In this study, we generated ORF67.5-deficient KSHV and its revertant. These viruses were then used to analyze the function of ORF67.5. ORF67.5-deficient KSHV was unable to produce infectious virions, and soccer ball-like capsids were mainly observed in infected cells. Furthermore, the ORF67.5-deficient KSHV lacked the ability to cleave the TR. Moreover, ORF67.5 promoted the interaction between ORF7 and ORF29, and the interaction between ORF67.5 and ORF7 was also increased by ORF29.

## RESULTS

### Construction of ORF67.5-deficient KSHV-bacterial artificial chromosome (BAC).

**FIG 1:**
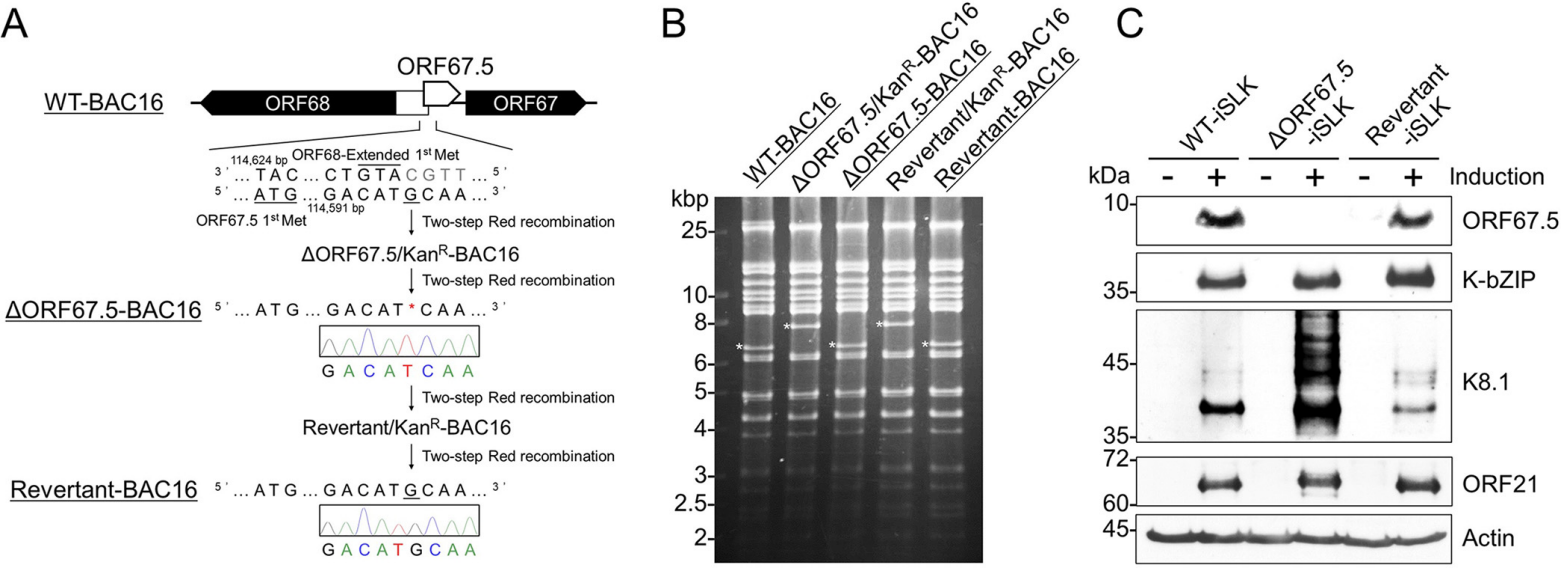
Construction of ΔORF67.5-BAC16 and Revertant-BAC16. (A) Diagram showing the location of KSHV ORF67.5 (nucleotides 114382 to 114624; accession number: GQ994935) and its neighboring ORFs on the KSHV-BAC (WT-BAC16). The adjacent DNA sequence of the mutation sites in ΔORF67.5-BAC16 and Revertant-BAC16 clones was confirmed by Sanger sequencing. (B) Agarose gel electrophoresis image of EcoRV-digested recombinant BAC16 clones. The asterisks indicate the insertion or deletion of a kanamycin resistance cassette in each BAC clone. (C) Western blot showing the disappearance of ORF67.5 expression in lytic-induced (+) or uninduced (−) ΔORF67.5-BAC16-harboring iSLK cells (ΔORF67.5-iSLK). WT-iSLK, ΔORF67.5-iSLK, and Revertant-iSLK were treated (or untreated) with Dox and SB for 72 h to induce lytic replication. An antibody against β-actin (actin) was used as a loading control. The lytic gene products, K-bZIP (DE), K8.1 (L), and ORF21 (L) were expressed in all cell lines, unlike ORF67.5, which was absent in ΔORF67.5-iSLK.

To analyze the function of KSHV ORF67.5, we constructed ORF67.5-deficient KSHV-BAC (ΔORF67.5-BAC16) and revertant KSHV-BAC (Revertant-BAC16). The KSHV genome contains several ORFs with overlapping coding regions. The N-terminal coding region of ORF67.5 has been reported to overlap with the N-terminal coding region of the ORF68 gene, which encodes a total length of 545 aa (36). However, Glaunsinger et al. (37) did not detect endogenous ORF68 with 545 aa in KSHV-infected cells. Instead, they only detected the 467-aa form of ORF68, in which the N-terminal 78-aa region is deleted (37). Thus, the 545-aa ORF68 has been described as ORF68-Extended (37). The start codon (the first methionine) of ORF68-Extended overlaps with the coding region of ORF67.5. To eliminate the effect of the ORF67.5 mutation on ORF68 expression, we deleted 1 bp upstream of the start codon in ORF68-Extended from wild-type KSHV-BAC to create ΔORF67.5-BAC16 (Fig. 1A). ΔORF67.5-BAC16 has a frameshift mutation (39th G-C bp deletion) at the N-terminal side of the ORF67.5 coding region, by which a nonsense mRNA is transcribed. This nonsense mRNA contains 12 aa from the N-terminal side of the ORF67.5 coding region (80 aa) followed by 11 aa unrelated to ORF67.5 and a stop codon. In addition to ΔORF67.5-BAC16, Revertant-BAC16 was constructed by reinsertion of the 1 bp deletion into ΔORF67.5-BAC16 (Fig. 1A). The sequence of the mutagenesis site and neighboring nucleotides of the constructed BACs were confirmed by Sanger sequencing (Fig. 1A). The insertion and removal of the kanamycin resistance cassette associated with mutagenesis was confirmed by EcoRV digestion of each BAC followed by agarose gel electrophoresis (Fig. 1B).

Wild-type BAC16, ΔORF67.5-BAC16, and Revertant-BAC16 were transfected into doxycycline-inducible ORF50-expressing SLK (iSLK) cells, and transfectants were selected with hygromycin B to establish WT-iSLK, ΔORF67.5-iSLK, and Revertant-iSLK cell lines, respectively. Here, each cell line is simply referred to as WT-iSLK, ΔORF67.5-iSLK, and Revertant-iSLK. Treatment of iSLK cells with doxycycline (Dox) and sodium butyrate (SB) induces KSHV lytic infection (38). To confirm the disappearance of full-length ORF67.5 protein expression in ΔORF67.5-iSLK, we generated a rabbit polyclonal antibody (pAb) against ORF67.5. Each cell line was treated with Dox and SB to induce lytic infection, and the cells were lysed. Because ORF67.5 is a low-molecular-weight (MW) protein, Western blotting was performed using tricine electrophoresis. In all cell lines, ORF67.5 expression was not detected when lytic infection was not induced. By contrast, when lytic infection was induced, ORF67.5 expression was confirmed in WT-iSLK and Revertant-iSLK but not in ΔORF67.5-iSLK (Fig. 1C). The predicted MW of the endogenous ORF67.5 is 9.2 kDa. This Western blot using the generated anti-ORF67.5 antibody did not detect nonspecific signals in the MW region below 15 kDa but did detect nonspecific signals in the higher MW regions. We also examined the expression of lytic gene products other than ORF67.5 by Western blotting. The DE gene product K-bZIP and the L gene product ORF21 were detected to the same extent in all induced iSLK cell lines (Fig. 1C). By contrast, the L gene product K8.1 was upregulated in ΔORF67.5-iSLK compared to in WT-iSLK and Revertant-iSLK (Fig. 1C). Although the reason for the increase in K8.1 expression is unknown, the same results were observed in multiple experiments. These data showed that ΔORF67.5-iSLK expressed the lytic gene products K-bZIP, K8.1, and ORF21 but not ORF67.5.

### ORF67.5 is required for infectious virus production.

**FIG 2:**
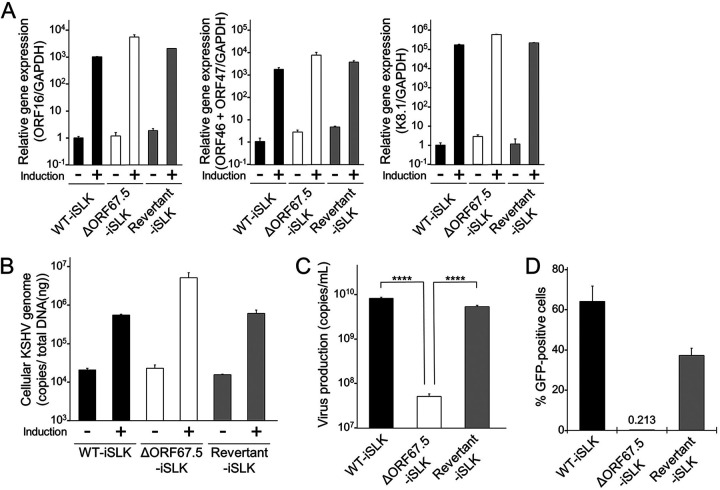
Characterization of ΔORF67.5 KSHV. (A) mRNA expression analysis of the following KSHV lytic genes: IE, ORF16; DE, ORF46 and ORF47; and L, K8.1. WT-iSLK, ΔORF67.5-iSLK, and Revertant-iSLK were treated with Dox and SB for 72 h to induce the lytic phase (+) or were uninduced (−). Total RNA was purified from harvested iSLK cells and subjected to RT-qPCR. The lytic gene mRNA levels were normalized to *GAPDH* mRNA levels. The values obtained from uninduced WT-iSLK were defined as 1.0. (B) Quantification of intracellular KSHV genomic DNA. WT-iSLK, ΔORF67.5-iSLK, and Revertant-iSLK were treated with Dox and SB to induce the lytic phase (+) or were uninduced (−). At 72 h postinduction, viral DNA was purified from the harvested cells. The intracellular viral genome copy number was then measured by qPCR and normalized to the amount of total DNA. (C) Quantification of extracellular encapsidated KSHV genomes. Each iSLK cell line was treated with Dox and SB for 72 h to induce the lytic phase, and the culture supernatants were harvested. Next, encapsidated KSHV genomic DNA from the culture supernatants was measured by qPCR; ****, *P* < 0.001. (D) Measurement of infectious virus. Each iSLK cell line was cultured with medium containing Dox and SB for 72 h to induce the lytic phase, and the culture supernatants were harvested. The supernatants were mixed with fresh HEK293T cells for infection with progeny virus. At 24 h postinfection, GFP-positive cells were counted by flow cytometry.

To elucidate the virological functions of ORF67.5, we examined the contribution of ORF67.5 to lytic gene expression during lytic infection. Each iSLK cell line was treated with Dox and SB to induce lytic infection, and the transcription levels of ORF16 (IE), ORF46 + ORF47 (DE), and K8.1 (L) were evaluated by reverse transcription-quantitative PCR (RT-qPCR). In all iSLK cell lines, we observed no remarkable differences in the mRNA expression levels of the tested lytic genes (Fig. 2A). In addition, qPCR measurement of the amount of intracellular viral genome replication showed no reduction of viral genomic DNA in ΔORF67.5-iSLK compared to in WT-iSLK and Revertant-iSLK (Fig. 2B). We also analyzed the effect of ORF67.5 deficiency on virus production and infectious virus production. qPCR-mediated quantification of the amount of DNase-resistant KSHV genomic DNA in the culture supernatant of lytic-induced cells showed that ΔORF67.5-iSLK produced significantly less virus than WT-iSLK and Revertant-iSLK (Fig. 2C). Next, the infectivity of the progeny virus produced by ΔORF67.5-iSLK was analyzed. The culture supernatant of lytic-induced ΔORF67.5-iSLK was coincubated with fresh HEK293T cells, and after 24 h, the percentage of green fluorescent protein (GFP)-positive HEK293T cells was measured by flow cytometry. Because BAC16 encodes the GFP gene, this supernatant transfer assay can assess the infectivity of the progeny virus by detecting GFP fluorescence in recipient cells (39). The infectivity of the progeny viruses produced in ΔORF67.5-iSLK was extremely low compared to those produced by WT-iSLK and Revertant-iSLK (Fig. 2D).

**FIG 3:**
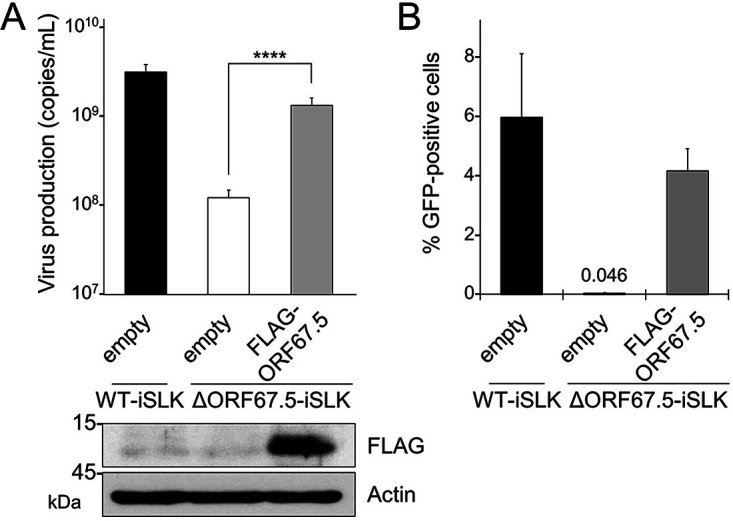
Complementation of ORF67.5-deficient KSHV with exogenous ORF67.5 expression. (A) Exogenous expression of ORF67.5 rescued the reduction in extracellular KSHV production observed in ΔORF67.5-iSLK. ΔORF67.5-iSLK was transiently transfected with empty plasmid (empty) or FLAG-ORF67.5 plasmid and was simultaneously cultured with medium containing Dox and SB for 72 h to induce the lytic phase. Encapsidated KSHV genomes in the culture supernatant were quantified by qPCR. Exogenous ORF67.5 expression was confirmed by Western blotting using an anti-FLAG (FLAG) antibody. An antibody against β-actin (actin) was used as a loading control; ****, *P* < 0.001. (B) Exogenous expression of ORF67.5 rescued the reduction in infectious virus production observed in ΔORF67.5-iSLK. ΔORF67.5-iSLK was transiently transfected with empty plasmid (empty) or FLAG-ORF67.5 expression plasmid and simultaneously cultured with medium containing Dox and SB for 72 h to induce the lytic phase. Harvested culture supernatants were then used for infection of fresh HEK293T cells. At 24 h postinfection, GFP-positive cells were counted by flow cytometry.

To further explore the involvement of ORF67.5 in virus production, we analyzed the complementation of virus production in ΔORF67.5-iSLK by exogenous ORF67.5 expression. ΔORF67.5-iSLK was transiently transfected with either empty plasmid or FLAG-tagged ORF67.5 expression plasmid, and the transfectants were treated with Dox and SB to induce lytic infection. The amount of virus production in the culture supernatant was quantified by qPCR. WT-iSLK transfected with empty plasmid was used as a control. The results showed that the reduced virus production in ΔORF67.5-iSLK was significantly complemented by the expression of exogenous ORF67.5 (Fig. 3A). Expression of exogenous ORF67.5 was confirmed by Western blotting with an antibody against FLAG (Fig. 3A, bottom). In addition, we examined the complementation of infectious virus production in ΔORF67.5-iSLK by exogenous ORF67.5 expression. Empty plasmid-transfected WT-iSLK and either empty plasmid- or FLAG-ORF67.5-transfected ΔORF67.5-iSLK were treated with Dox and SB to induce lytic infection. Next, the infectivity of the progeny virus in the culture supernatant was evaluated by a supernatant transfer assay. We found that exogenous expression of ORF67.5 overcame the reduction in infectious virus production by the ORF67.5-deficient cells ΔORF67.5-iSLK (Fig. 3B). These results indicate that ORF67.5 is dispensable for lytic gene transcription and viral genome replication, but it is required for virus production and infectious virus production.

### ORF67.5-deficient KSHV forms soccer ball-like capsids.

**FIG 4:**
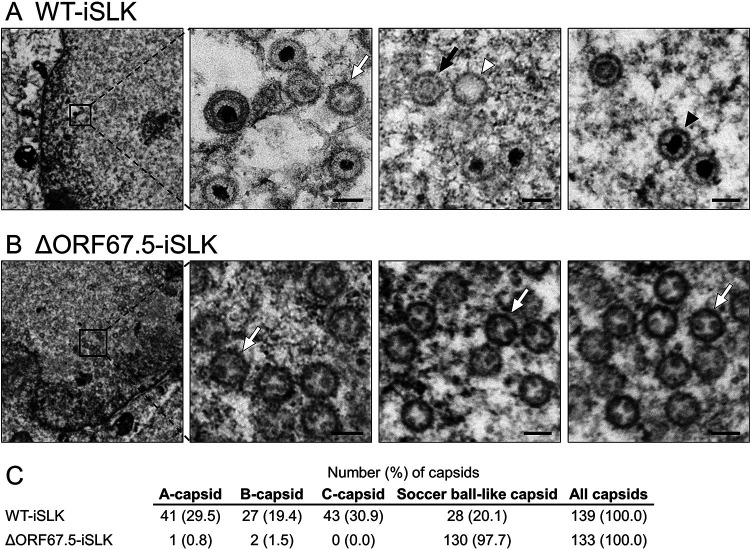
TEM images showing capsids formed in WT-iSLK and ΔORF67.5-iSLK. (A and B) Cells were lytically induced for 48 h, and nuclear viral capsids in WT-iSLK (A) and ΔORF67.5-iSLK (B) were observed by TEM. The black arrowheads, white arrowheads, black arrows, and white arrows indicate C-capsids, A-capsids, B-capsids, and soccer ball-like capsids, respectively. Scale bars, 100 nm. (C) Quantification of each capsid type observed by TEM.

KSHV ORF7 and ORF67.5 can be presumed to be components of the KSHV terminase complex. We previously reported that in ORF7-deficient KSHV-harboring cells, most capsids formed in the nucleus during lytic infection were immature capsids, which resembled the telstar pattern of a soccer ball (32). Therefore, we referred to these capsids as “soccer ball-like capsids” (32). In this study, we analyzed capsid formation in lytic-induced ORF67.5-deficient KSHV-harboring cells. WT-iSLK and ΔORF67.5-iSLK were treated with Dox and SB for 48 h to induce lytic infection, and the morphology of capsids formed in the nuclei was observed by TEM. WT-iSLK produced all types of capsids, including A-capsids, B-capsids, C-capsids, and soccer ball-like capsids (Fig. 4A). By contrast, mature capsids (C-capsids) were not detected in ΔORF67.5-iSLK, and most of the capsids formed were soccer ball-like capsids (Fig. 4B). The quantification of each capsid type is shown in Fig. 4C. Our results indicate that ORF67.5 contributes to mature capsid formation, and lack of ORF67.5 results in accumulation of soccer ball-like capsids, which is also observed in lytically induced ORF7-deficient KSHV-harboring cells.

### ORF67.5 is required for TR cleavage of the KSHV genome.

**FIG 5:**
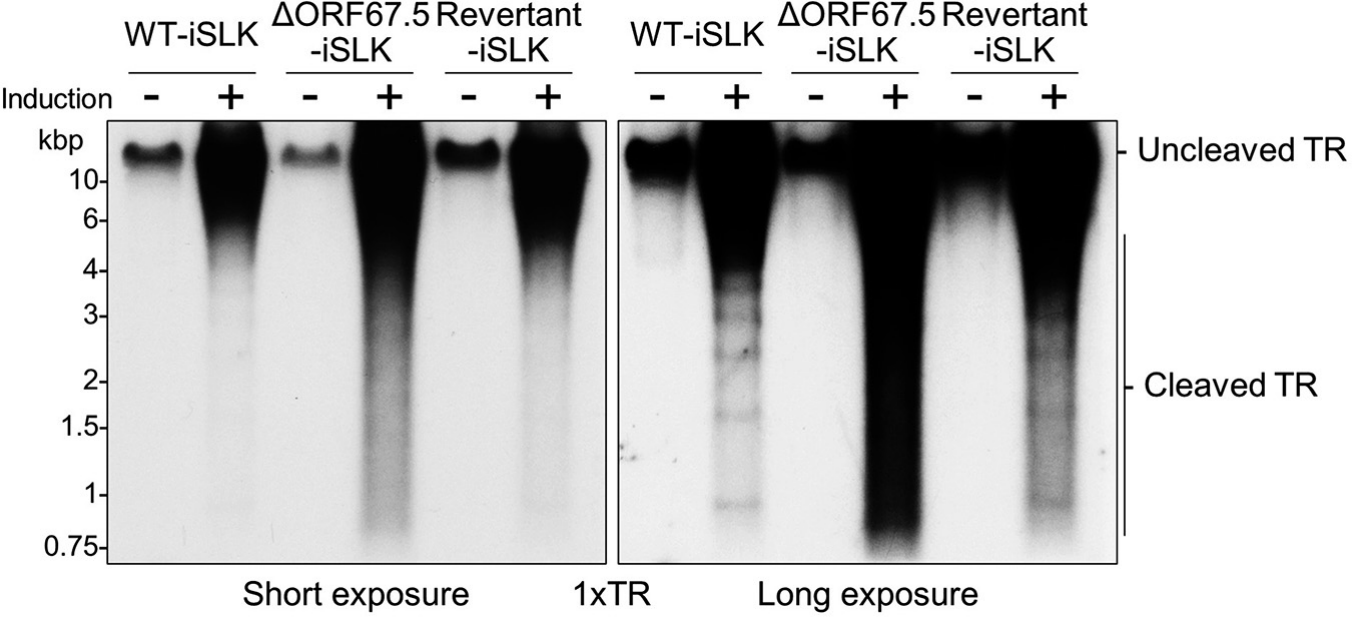
ΔORF67.5-KSHV failed to cleave the TR sites in the viral genome. WT-iSLK, ΔORF67.5-iSLK, and Revertant-iSLK were treated with (+) or without (−) Dox and SB for 72 h to induce the lytic phase. Purified total genomic DNA was digested with EcoRI and SalI and subjected to Southern blotting. Uncleaved and cleaved TRs were detected using a DIG-labeled 1×TR probe, and their migration patterns are denoted to the right of the images; left, short exposure; right, long exposure.

We analyzed the contribution of ORF67.5 in TR cleavage of the replicated KSHV genome during lytic infection. The cleaved TR fragments were detected by a method established by Glaunsinger et al. with some modifications (the same method by which we previously analyzed ΔORF7-KSHV) (32, 37). ΔORF67.5-iSLK was treated with Dox and SB for 72 h to induce lytic infection. Next, the total genomic DNA was digested with restriction enzymes and subjected to Southern blotting using a probe against 1×TR to detect viral DNA fragments containing TRs. Uncleaved TR-containing DNA was detected in all cell lines and was increased in all lytic-induced cell lines compared to in uninduced cell lines (Fig. 5). These data showed that KSHV genome replication in ΔORF67.5-iSLK was similar to that observed in WT-iSLK, supporting the results shown in Fig. 2B (i.e., ORF67.5 is dispensable for viral genome replication) (Fig. 5). In contrast to the uncleaved TR, multiple cleaved TR fragments were detected in lytic-induced WT-iSLK and Revertant-iSLK but not in lytic-induced ΔORF67.5-iSLK (Fig. 5). These fragment patterns were consistent with those in our previous report of ΔORF7-KSHV and in the report of ΔORF68-KSHV by Glaunsinger et al. (32, 37). These results demonstrate that ORF67.5 is required for TR cleavage of the KSHV genome.

### The conserved regions of ORF67.5 are important for virus production.

**FIG 6:**
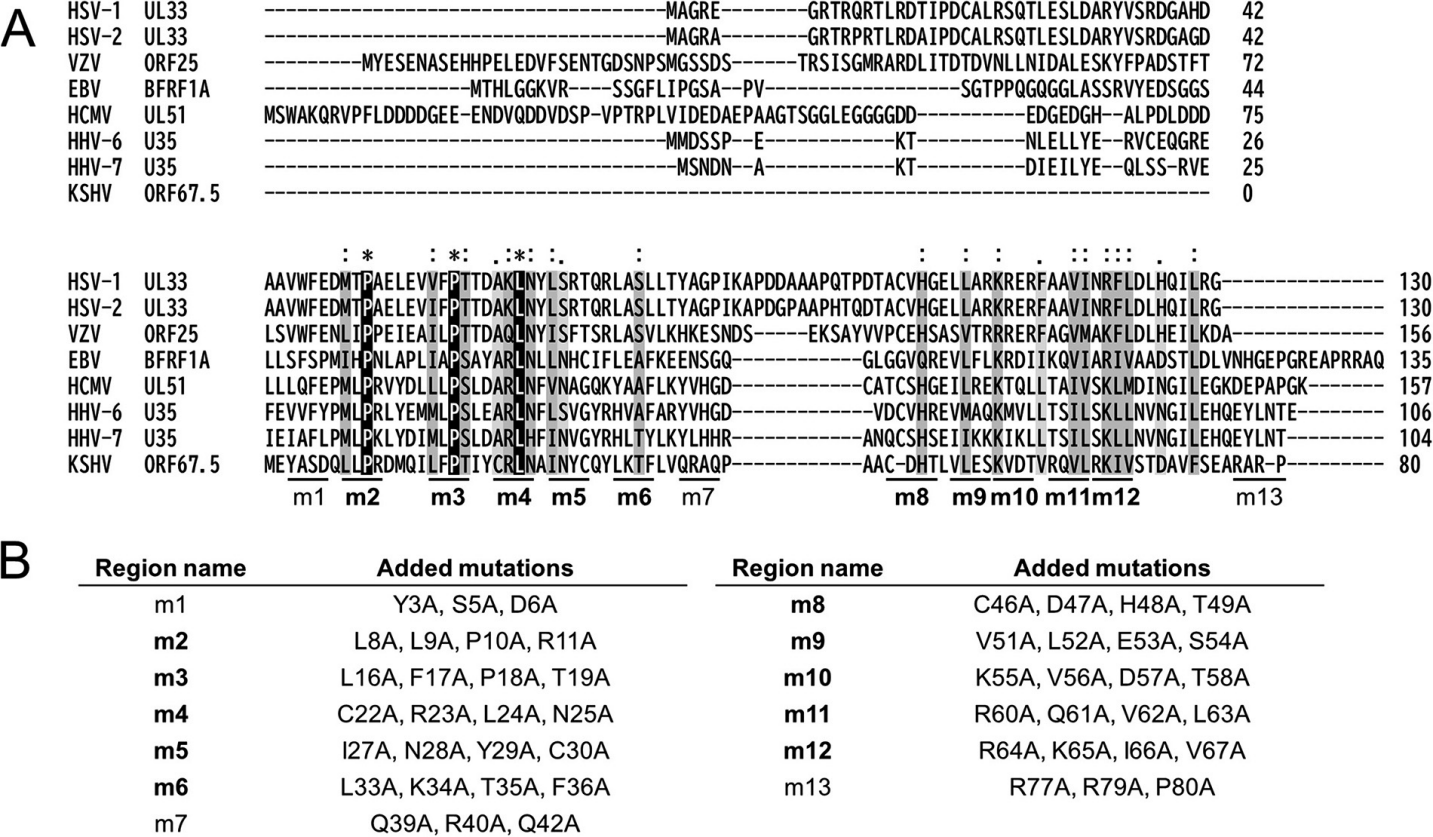
Amino acid sequence alignment between KSHV ORF67.5 and its human herpesviral homologs as well as construction of the ORF67.5 alanine mutants. (A) Amino acid sequences were obtained from the NCBI database (HSV-1 UL33 [GenBank: QAU10292], HSV-2 UL33 [NCBI Reference Sequence: YP_009137185], VZV ORF25 [GenBank: QXM18354], EBV BFRF1A [GenBank: AAA45868], HCMV UL51 [GenBank: QHB20503], HHV-6 U35 [NCBI Reference Sequence: NP_042928], HHV-7 U35 [GenBank: AAC40749], and KSHV ORF67.5 [GenBank: QFU18781]). The raw data used for the alignment were obtained with the Clustal Omega program (EMBL-EBI; https://www.ebi.ac.uk/Tools/msa/clustalo/). The background indicates the degree of homology. The completely matched amino acid residues are shown in white letters with a black background, and similar amino acid residues are shown in black letters with a gray background. The conserved regions (m2 to 6 and m8 to 12) and the nonconserved regions (m1, m7, and m13) among the human herpesviral ORF67.5 homologs are indicated at the bottom of the sequence alignments, and the conserved regions are indicated in bold. (B) The amino acid sequences of the conserved and nonconserved regions of the human herpesviral ORF67.5 homologs that were substituted with alanine in each ORF67.5 mutant. The names of the conserved regions are indicated in bold.

To obtain further information on the functional significance of the KSHV ORF67.5 protein, we aligned the amino acid sequences of the human herpesviral ORF67.5 homologs. Figure 6A shows the amino acid sequence alignments of KSHV ORF67.5 homologs from HSV-1, HSV-2, VZV, EBV, HCMV, HHV-6, and HHV-7. The amino acid residues that completely match are shown in white letters with a black background, and similar amino acid residues are shown in black letters with a gray background. This analysis revealed the conserved regions within the KSHV ORF67.5 protein (Fig. 6A). Interestingly, KSHV ORF67.5 has the shortest amino acid sequence among its homologs. To determine the functionally important region(s) of ORF67.5 that contribute to virus production, we generated a total of 13 alanine mutants spanning the conserved regions (m2 to 6 and m8 to 12) and nonconserved regions (m1, m7, and m13) of ORF67.5 (Fig. 6B). In this report, each mutated region in ORF67.5 and each ORF67.5 alanine mutant are referred to as m1 to 13 and ORF67.5 m1 to 13, respectively (Fig. 6).

**FIG 7:**
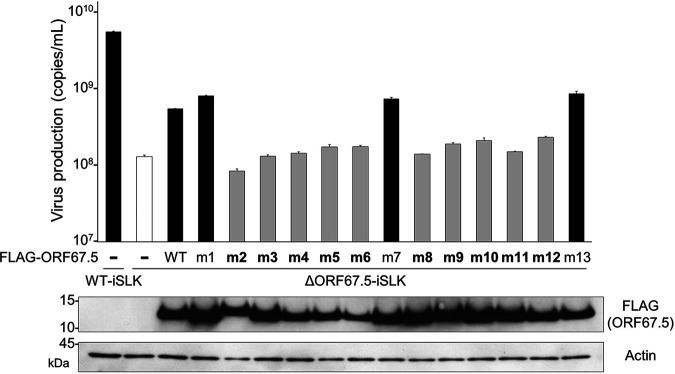
The ORF67.5 conserved region alanine mutants failed to rescue the reduction in virus production from ΔORF67.5-iSLK. To evaluate the contribution of the conserved and nonconserved regions of ORF67.5 to virus production, complementation assays were performed by exogenous ORF67.5 alanine mutant expression in ΔORF67.5-iSLK. ΔORF67.5-iSLK was transiently transfected with FLAG-ORF67.5 WT or FLAG-ORF67.5 alanine mutant expression plasmids (FLAG-ORF67.5 m1 to m13). During transfection, the cells were simultaneously cultured in medium containing Dox and SB for 72 h to induce the lytic phase. Encapsidated KSHV genomes in the culture supernatants were quantified by qPCR. Expression of exogenous FLAG-tagged ORF67.5 WT and mutants was confirmed by Western blotting using an anti-FLAG (FLAG) antibody.

We then used these mutants to analyze the impact of the KSHV ORF67.5 conserved regions on virus production (Fig. 7). To this end, we conducted a complementation assay to determine if any of the ORF67.5 alanine mutant (m1 to 13) expression plasmids could rescue virus production in ΔORF67.5-iSLK. ΔORF67.5-iSLK was transfected with each alanine mutant plasmid, and lytic infection was induced with Dox and SB. The culture supernatants were harvested 72 h after induction, and virus production in the supernatants was measured by qPCR. The low virus production in ΔORF67.5-iSLK was complemented by transfection of the ORF67.5 wild-type (WT) expression plasmid (Fig. 7), which is also shown in Fig. 3A. However, among the ORF67.5 alanine mutants, only ORF67.5 m1, m7, and m13 restored the reduction in virus production from ΔORF67.5-iSLK, while the other mutants failed to restore it (Fig. 7). The expression of each ORF67.5 mutant was confirmed by Western blotting (Fig. 7, bottom). These results indicate that the m2 to 6 and m8 to 12 regions in ORF67.5 are essential for the function of ORF67.5 in virus production, whereas the m1, m7, and m13 regions in ORF67.5 are not essential for the function of ORF67.5 in virus production. Importantly, the m2 to 6 and m8 to 12 regions within the ORF67.5 protein are highly conserved among herpesviruses, whereas the m1, m7, and m13 regions are not conserved.

### The conserved regions of ORF67.5 are important for its interaction with ORF7.

**FIG 8:**
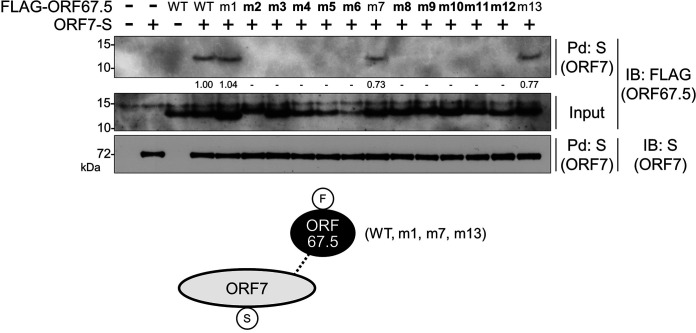
The conserved regions of ORF67.5 were necessary for the interaction of ORF67.5 with ORF7. An expression plasmid encoding FLAG-tagged wild-type ORF67.5 (FLAG-ORF67.5 WT) or FLAG-tagged ORF67.5 mutant (FLAG-ORF67.5 m1 to m13) was cotransfected with an S-tagged ORF7 expression plasmid (ORF7-S) into HEK293T cells. Next, the cell lysates were subjected to pulldown assays (Pd) using S-protein immobilized beads (S) for the precipitation of ORF7-S protein. Any FLAG-ORF67.5 protein that interacted with ORF7 was detected by Western blotting using an anti-FLAG antibody (FLAG). The proteins in parentheses indicate which protein was recognized by the assay. The band intensities of the FLAG-ORF67.5 are shown at the bottom of the image. The intensity value of FLAG-ORF67.5 in the cell transfected with FLAG-ORF67.5 WT and ORF7-S was defined as 1.0. When the intensity value was the less than 0.01, “−” was indicated. The conserved region mutants of ORF67.5 are indicated in bold. Bottom, schematic representation of the results with the protein tags indicated in the circles; IB, immunoblot.

We previously reported that a pulldown assay did not detect a direct interaction between ORF67.5 and ORF29, but a direct interaction between ORF67.5 and ORF7 was detected (31). Furthermore, we showed above that the m2 to 6 and m8 to 12 regions in the ORF67.5 protein, which are conserved among herpesviral ORF67.5 homologs, are important for the virus-producing function of ORF67.5 (Fig. 6 and 7). Therefore, we tested whether the conserved regions in ORF67.5 contribute to its direct interaction with ORF7. To this end, pulldown assays were performed using the ORF67.5 m1 to 13 alanine mutants. Each FLAG-tagged ORF67.5 alanine mutant plasmid was cotransfected together with an S-tagged ORF7 plasmid into HEK293T cells. Next, S-tagged ORF7 was precipitated with S-protein agarose to detect the interaction between ORF7 and each ORF67.5 mutant. Our results showed that ORF67.5 WT interacted with ORF7, and the ORF67.5 m1, m7, and m13 mutants also interacted with ORF7 (Fig. 8). However, the ORF67.5 m2 to 6 and m8 to 12 mutants failed to interact with ORF7 (Fig. 8). These data suggest that the conserved regions (m2 to 6 and m8 to 12) in the ORF67.5 protein are involved in both virus production and the interaction of ORF67.5 with ORF7. In addition, we need to consider the possibility that the alanine substitution in these mutants affects the proper folding or maintenance of the protein structure of ORF67.5.

### ORF67.5 promotes the interaction between ORF7 and ORF29, and the interaction between ORF67.5 and ORF7 is increased by ORF29.

**FIG 9:**
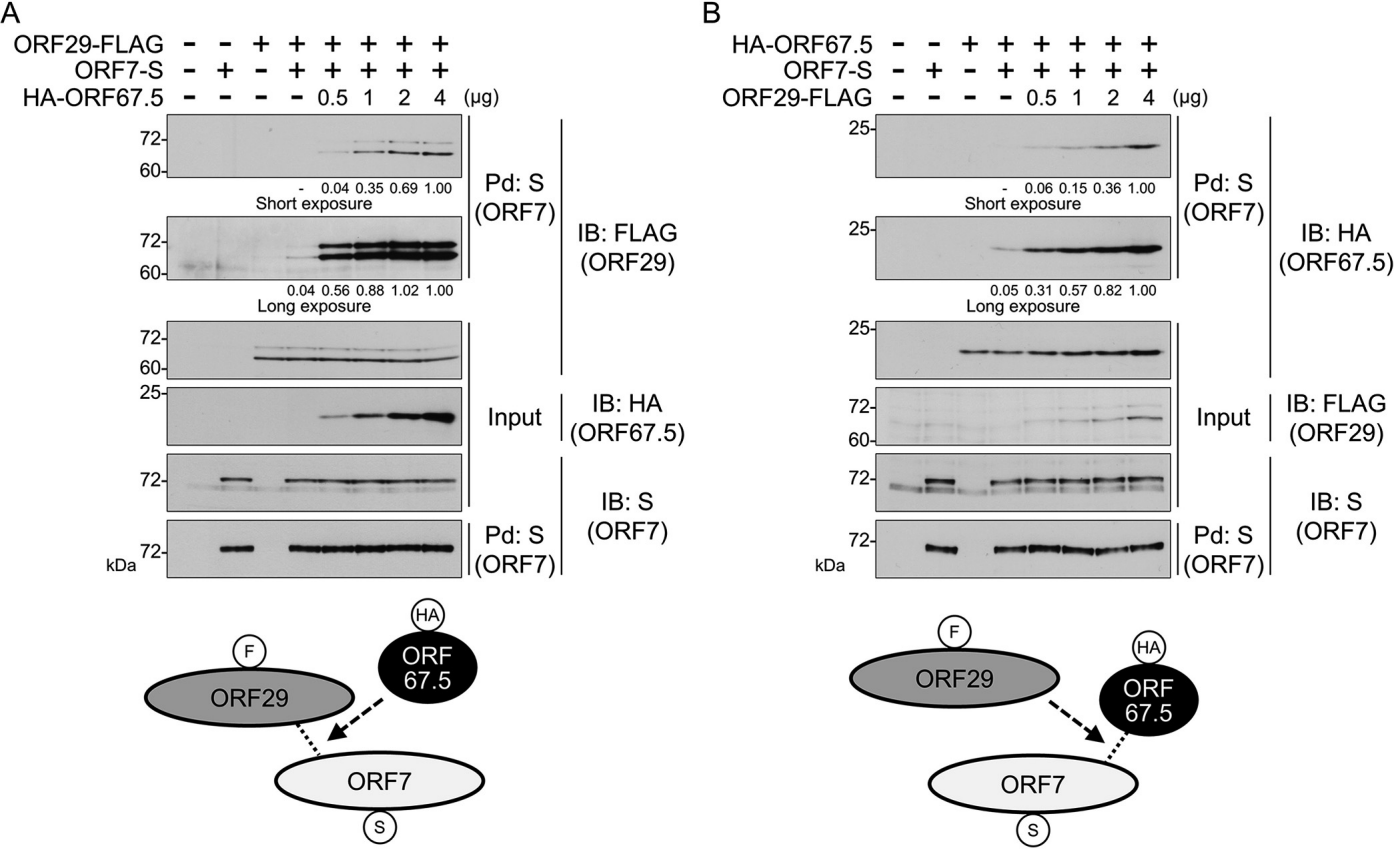
ORF67.5 promoted the interaction between ORF7 and ORF29, and ORF29 increased the interaction between ORF67.5 and ORF7. (A) Expression plasmids encoding ORF29-FLAG and ORF7-S were cotransfected with various amounts of HA-ORF67.5 expression plasmid into HEK293T cells. Next, the cell lysates were subjected to pulldown (Pd) assays using S-protein immobilized beads (S) for the precipitation of ORF7-S protein. Any ORF29-FLAG protein that interacted with ORF7 was detected by Western blotting with an anti-FLAG antibody (FLAG). The proteins in parentheses indicate which protein was recognized by the assay. The band intensities of ORF29-FLAG are shown at the bottom of the image, and the value of ORF29-FLAG in the cell transfected with ORF29-FLAG, ORF7-S, and HA-ORF67.5 (4 μg) is defined as 1.0. When the intensity value was the less than 0.01, “−” was indicated. Bottom, schematic representation of the results with the protein tags indicated in the circles. (B) Expression plasmids encoding HA-ORF67.5 and ORF7-S were cotransfected with various amounts of ORF29-FLAG expression plasmid into HEK293T cells. Next, the cell lysates were subjected to pulldown (Pd) assays using S-protein immobilized beads (S), followed by Western blotting with anti-HA antibody (HA) to detect the ORF7-binding HA-ORF67.5 protein. The band intensities of HA-ORF67.5 are shown at the bottom of the image. The value of HA-ORF67.5 in the cell transfected with HA-ORF67.5, ORF7-S, and ORF29-FLAG (4 μg) was defined as 1.0. When the intensity value was the less than 0.01, “−” was indicated.

ORF7 has been reported to interact with both ORF29 and ORF67.5, although a direct interaction between ORF67.5 and ORF29 was not detected in pulldown assays (31). Therefore, we investigated the effect of ORF67.5 on the interaction between ORF7 and ORF29. When FLAG-tagged ORF29 and S-tagged ORF7 were cotransfected into HEK293T cells, ORF7 interacted with ORF29 (Fig. 9A). Remarkably, cotransfection of hemagglutinin (HA)-tagged ORF67.5 increased the interaction between ORF7 and ORF29 in an ORF67.5 expression-level-dependent manner (Fig. 9A). The Western blotting data of the input samples showed that the presence of ORF67.5 had no effect on ORF7 or ORF29 expression levels (Fig. 9A). These results revealed that ORF67.5 promotes the interaction between ORF7 and ORF29.

Next, we examined the effect of ORF29 on the interaction between ORF7 and ORF67.5. When HA-tagged ORF67.5 and S-tagged ORF7 were cotransfected into HEK293T cells, ORF7 interacted with ORF67.5 (Fig. 9B). As expected, cotransfection of FLAG-tagged ORF29 increased the interaction between ORF7 and ORF67.5 in an ORF29 expression-level-dependent manner (Fig. 9B). The Western blotting data of the input samples showed that the presence of ORF29 had no effect on ORF7 or ORF67.5 expression levels (Fig. 9B). These results showed that the interaction between ORF67.5 and ORF7 is increased by ORF29.

### Identification of the regions in ORF67.5 responsible for promoting the interaction between ORF7 and ORF29.

**FIG 10:**
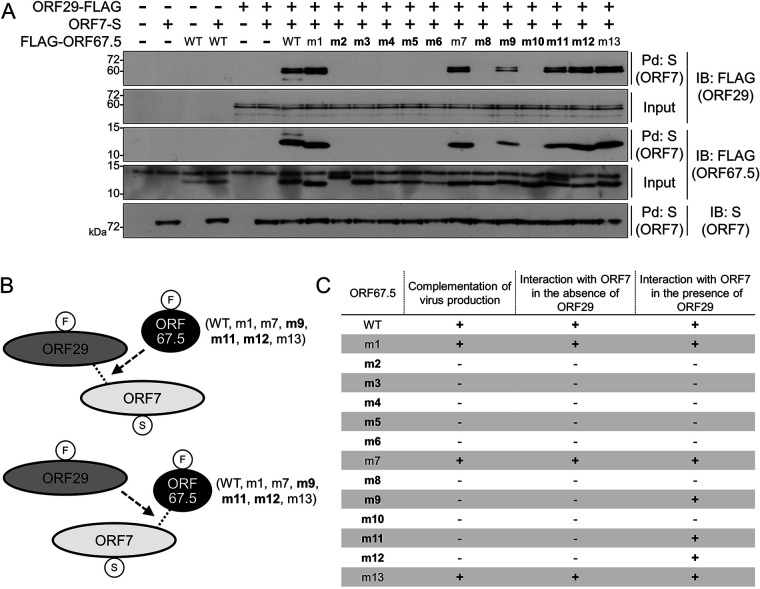
The effects of the conserved and nonconserved region mutants of ORF67.5 on the interaction between ORF7 and ORF29 as well as the effect of ORF29 on the interaction between each ORF67.5 mutant and ORF7. (A) Expression plasmids encoding ORF29-FLAG and ORF7-S were cotransfected with the FLAG-ORF67.5 mutant expression plasmid into HEK293T cells. Next, the cell lysates were subjected to pulldown (Pd) assays using S-protein immobilized beads (S) followed by Western blotting using anti-FLAG or anti-S antibodies (FLAG and S, respectively). Any ORF29-FLAG protein that interacted with ORF7-S in the presence of wild-type (WT) or alanine mutant ORF67.5 (m1 to m13) was detected by Western blotting with anti-FLAG antibody. The proteins in parentheses indicate which protein was recognized by the assay. (B) Schematic representation of the interaction relationship among ORF7, ORF29, and ORF67.5 in this experiment. The protein tags are indicated in the circles, and the conserved region mutants of ORF67.5 are shown in bold. (C) A table summarizing the characteristics of the wild-type (WT) and alanine mutants of ORF67.5 (m1 to m13) revealed by our experiments. The table summarizes the results obtained in Fig. 7 (complementation of virus production), Fig. 8 (the interaction with ORF7 in the absence of ORF29), and Fig. 10A (the interaction with ORF7 in the presence of ORF29). The conserved region mutants in ORF67.5 are indicated in bold.

We disclosed that the conserved region mutants (m2 to 6 and m8 to 12) of ORF67.5 failed to rescue the reduction in virus production from ΔORF67.5-iSLK (Fig. 7) and failed to interact with ORF7 (Fig. 8). In other words, these regions, which are highly conserved among human herpesvirus homologs, are important for virus production and the interaction between ORF67.5 and ORF7. In addition, we found that the interaction between ORF7 and ORF29 was increased by ORF67.5, and the interaction between ORF7 and ORF67.5 was increased by ORF29 (Fig. 9). Therefore, we investigated whether the interaction between ORF7 and ORF29 was increased by each ORF67.5 mutant (Fig. 10A and B). Moreover, we determined whether the interaction between ORF7 and each ORF67.5 mutant was increased by ORF29 (Fig. 10A and B). As expected, the interaction between ORF7 and ORF29 was increased by the WT ORF67.5 and the nonconserved region mutants (ORF67.5 m1, m7, and m13), which rescued virus production in ΔORF67.5-iSLK, as shown in Fig. 7. The interaction of ORF7 with the nonconserved region mutants (ORF67.5 m1, m7, and m13) was also increased by ORF29 (Fig. 10A and C). Interestingly, in addition to the nonconserved region mutants (ORF67.5 m1, m7, and m13), the conserved region mutants (ORF67.5 m9, m11, and m12) also increased the interaction between ORF7 and ORF29. Furthermore, the interaction of ORF7 with the conserved region mutants (ORF67.5 m9, m11, and m12) was increased by ORF29 (Fig. 10A and C). However, these conserved region mutants (ORF67.5 m9, m11, and m12) could not overcome the reduction in virus production from ΔORF67.5-iSLK (Fig. 7) and did not have the ability to interact with ORF7 in the absence of ORF29 (Fig. 8). Moreover, the conserved region mutants (ORF67.5 m2, m3, m4, m5, m6, m8, and m10) failed to interact with ORF7 neither in the absence (Fig. 8) nor in the presence (Fig. 10) of ORF29 and failed to increase the interaction between ORF7 and ORF29. Based on these findings, the nonconserved regions (m1, m7, and m13) of ORF67.5 had no effect on virus production nor the promotion of the interaction between ORF7 and ORF29. By contrast, the conserved regions (m9, m11, and m12) of ORF67.5 were required for its effects on virus production and for the interaction of ORF67.5 with ORF7 in the absence of ORF29. However, these conserved m9, m11, and m12 regions of ORF67.5 were not required for the interaction between ORF67.5 and ORF7 in the presence of ORF29 nor for the ORF67.5-mediated promotion of the interaction between ORF7 and ORF29. Because the conserved regions (m2 [L8, L9, P10, and R11], m3 [L16, F17, P18, and T19], m4 [C22, R23, L24, and N25], m5 [I27, N28, Y29, and C30], m6 [L33, K34, T35, and F36], m8 [C46, D47, H48, and T49], and m10 [K55, V56, D57, and T58]) of ORF67.5 contribute to both virus production and the interaction of ORF67.5 with ORF7 (regardless of the presence or absence of ORF29), these regions are critically important for ORF67.5 function.

## DISCUSSION

In this study, we showed that KSHV ORF67.5 was essential for infectious virus production, normal capsid formation, and TR cleavage of viral genomes. In addition, ORF67.5 enhanced the interaction between ORF7 and ORF29, and ORF29 also increased the interaction between ORF67.5 and ORF7. These results suggest that the KSHV terminase complex is composed of ORF7, ORF29, and ORF67.5, which are the KSHV homologs of the HSV-1 terminase complex components UL28, UL15, and UL33, respectively. ORF67.5 facilitates the formation of the ORF7-ORF29-ORF67.5 complex and is crucial for the functional activity of the terminase complex, including TR cleavage. In addition, the conserved regions (m2 to 6 and m8 to 12) of ORF67.5 are important for virus production. Additionally, m2 to 6, m8, and m10 are required not only for virus production but also for the ORF67.5-mediated promotion of the interaction between ORF7 and ORF29. To our knowledge, this is the first report describing the virological significance and functions of ORF67.5 as a component of the KSHV terminase complex.

Numerous soccer ball-like capsids were observed in lytic-induced ΔORF67.5-iSLK (Fig. 4B). Capsids similar in morphology to the soccer ball-like capsids have also been observed in VZV and KSHV (32, 40–44). The soccer ball-like capsid is thought to be a state in which degraded scaffold proteins remain in the capsid after procapsid formation (32, 40). Similar to KSHV ORF67.5, deletion of KSHV ORF7, which is hypothesized to be one of the components of the KSHV terminase complex, also resulted in the formation of soccer ball-like capsids (32). The HSV-1 terminase complex excises and packages one unit of the viral genome from serially replicated viral genome precursors. During lytic replication, soccer ball-like capsid formation occurs when the ORF7- or ORF67.5-defective KSHV-harboring cell exhibits a defect in TR cleavage of the viral genome. These findings suggest that the KSHV capsid formation process may be arrested at the soccer ball-like capsid stage when viral genome precursor cleavage or genome packaging fails. During the KSHV capsid maturation process, when the inner scaffold layer of the procapsid is detached from the outer capsid by the viral protease ORF17, the degradation and disruption of the scaffold inner structure is proceeded (45). If the soccer ball-like capsids contain residual products derived from the disrupted inner scaffold structure, the terminase complex would not be required for the disruption (or degradation) of the scaffold proteins within the capsid. However, the external release of the disrupted scaffold structure may either require an unknown novel function of the KSHV terminase complex or insertion of the KSHV genome into the capsid. In other words, the collapsed scaffold proteins may be eliminated from the capsid through gaps in the outer capsid layer when these residual scaffold proteins are pushed out by the insertion of the KSHV genome. This hypothesis has also been proposed by Zhou et al. (40). Based on this hypothesis, when ORF7 or ORF67.5 is defective, the insertion of the KSHV genome into the capsid does not occur, and the collapsed scaffold inner structures are not eliminated from the capsid. Thus, the capsid formation process may be arrested at the soccer ball-like capsid stage. When herpesvirus genome insertion occurs, the state of the capsid is still unclear, but further development of observational techniques, such as time-lapse cryo-electron microscopy (cryo-EM), will be required to confirm the state in real time (11, 46).

KSHV ORF67.5 has the shortest amino acid sequence among its human herpesviral homologs (Fig. 6A). The KSHV ORF67.5 homolog, HSV-1 UL33, has an additional 42 amino acids on the N-terminal side, which are not present in KSHV ORF67.5. The N-terminal region of HSV-1 UL33 does not contribute to virus production, and its biological function is unknown (47). The m2 to 6 and m8 to 12 regions are highly conserved among the human herpesviral KSHV ORF67.5 homologs (Fig. 6A). The conserved region mutants of KSHV ORF67.5 (ORF67.5 m2 to 6 and m8 to 12) could not complement the reduction in virus production from ΔORF67.5-iSLK (Fig. 7). Further, we did not detect the interaction of ORF7 with these mutants in the absence of ORF29 (Fig. 8). By contrast, the nonconserved region mutants of KSHV ORF67.5 (ORF67.5 m1, m7, and m13) complemented virus production (Fig. 7) and interacted with ORF7 (Fig. 8). These findings suggest that the important regions within ORF67.5 that are involved in the formation and function of the KSHV terminase complex are highly conserved among human herpesviral homologs.

**FIG 11:**
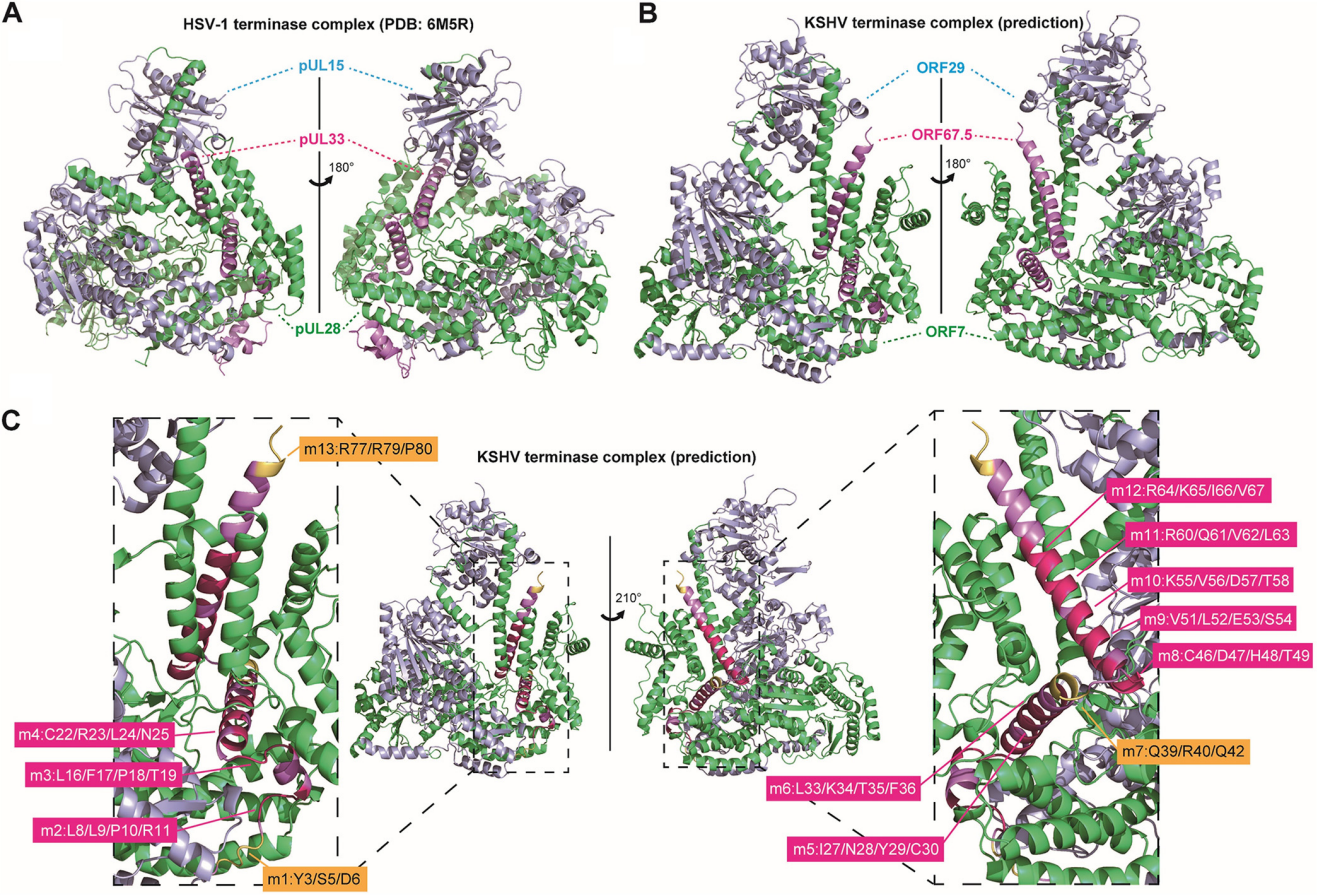
The structure of the HSV-1 terminase complex and the predicted structure model of the KSHV terminase complex. (A) The HSV-1 monomeric terminase complex structure (PDB: 6M5R) (21). Each HSV-1 protein cartoon was colored as follows: HSV-1 pUL28 (green), pUL15 (light blue), and pUL33 (light purple). Unmodelled amino acid residues, expected to comprise the unfolded region of each protein, were listed as follows: HSV-1 pUL28 (aa 268 to 305, 433 to 477, and 652 to 653), pUL15 (aa 178 to 194, 603 to 613, and 686 to 704), and pUL33 (aa 83 to 95). (B) The KSHV monomeric terminase complex was predicted by AlphaFold2. Each KSHV protein cartoon was colored as follows: KSHV ORF7 (green), ORF29 (light blue), and ORF67.5 (light purple). The KSHV terminase complex proteins were colored according to their HSV-1 homologs. (C) Based on the predicted KSHV terminase complex model, the main chain cartoon of the KSHV ORF67.5 regions substituted with alanine in the present study was visualized with rotation. The mutated regions (m1, m7, and m13), which are dispensable for the function of ORF67.5 in virus production, are highlighted in yellow. The mutated regions (m2 to 6 and m8 to 12) that are essential for the function of ORF67.5 in virus production are highlighted in magenta.

To further discuss the functionally critical regions of KSHV ORF67.5, the conformation of the tripartite complex comprising ORF7, ORF29, and ORF67.5 was predicted using the artificial intelligence (AI) deep learning algorithm AlphaFold2 (Fig. 11). The conformation of the HSV-1 terminase complex composed of HSV-1 UL28 (green), UL15 (light blue), and UL33 (light purple) has already been determined using cryo-EM (21). We compared this conformation with the AI-predicted tripartite complex conformation of ORF7 (green), ORF29 (light blue), and ORF67.5 (light purple) (Fig. 11A and B). The overall conformation was largely consistent between the HSV-1 terminase complex and the putative KSHV terminase complex, with several differences. In the KSHV ORF7, ORF29, and ORF67.5 tripartite complex, ORF67.5 appeared to be wrapped in ORF7 and was not associated with ORF29. From another point of view, the ORF7-interacting ORF67.5 resembled a wheel clamp that supported the conformation of ORF7. These AI-predicted conformational data generally supported the results obtained by the pulldown assay (i.e., ORF67.5 interacted with ORF7 but not with ORF29) (31). It should be noted that in the actual HSV-1 terminase complex, tripartite complexes composed of UL28, UL15, and UL33 form a hexameric ring structure (21). KSHV ORF67.5 has three intramolecular helical structures, and its N- and C-terminal regions were hypothesized to fluctuate freely and not form a characteristic secondary structure (Fig. 11B). The m1 to 13 regions of ORF67.5 analyzed in this paper are indicated in this prediction model. In ORF67.5, the nonconserved region m7 is located at the junction of the helical structure, and the nonconserved regions m1 and m13 are located at the N-terminal and C-terminal regions, which were hypothesized to fluctuate freely (Fig. 11C). The conserved regions m2 to 6 and m8 to 12 are all located in the helical structure of ORF67.5 (Fig. 11C). These models suggest that the formation of the helical structures is likely important for ORF67.5 function and may explain why the amino acid sequences of the helical structure-forming regions are highly conserved. This explanation is supported by our data, showing that mutations in the conserved regions (m2 to 6 and m8 to 12) of ORF67.5 impaired virus production (Fig. 7). This structural model is a prediction model, and the three-dimensional structure of the actual KSHV terminase complex needs to be elucidated by cryo-EM or X-ray structure analysis.

The interaction between ORF7 and ORF29 was promoted by ORF67.5, and the interaction between ORF67.5 and ORF7 was enhanced by ORF29 (Fig. 9A and B). In addition, we detected neither an ORF67.5-dependent increase in ORF7 and ORF29 expression nor an ORF29-dependent increase in ORF67.5 and ORF7 expression (Fig. 9A and B). These data indicate that the increase in each protein-protein interaction is not due to an increase in expression or stabilization of the two interacting proteins. Instead, the mechanism involves the enhancement or stabilization of the protein-protein interactions. As for the HSV-1 terminase complex components, UL28 (KSHV ORF7 homolog) directly interacts with UL15 (KSHV ORF29 homolog) and UL33 (KSHV ORF67.5 homolog) (21, 22). Similar to KSHV ORF67.5, UL33 increases the interaction between UL28 and UL15. However, unlike KSHV ORF29, UL15 has no effect on the interaction between UL33 and UL28 (22). These results show that HSV-1 and KSHV homologs share some common features (and some differences). Specifically, the interaction between UL28 (ORF7) and UL15 (ORF29) is supported by UL33 (ORF67.5). On the other hand, in HSV-1, the interaction between UL33 and UL28 is not supported by UL15, but in KSHV, the interaction between ORF67.5 and ORF7 was increased in an ORF29 expression-level-dependent manner. In addition, it has been reported that HSV-1 UL28 protects UL33 from proteasomal degradation (22), whereas our data showed that ORF67.5 was not stabilized by ORF7 (Fig. 8).

The ORF67.5 nonconserved region mutants (ORF67.5 m1, m7, and m13) retained the ability to complement the reduced virus production caused by ORF67.5 deletion and retained the ability to interact with ORF7 in the absence of ORF29 (Fig. 7 and 8). However, all the conserved region mutants of ORF67.5 (ORF67.5 m2 to 6 and m8 to 12) lost both the ability to complement virus production and the ability to interact with ORF7 in the absence of ORF29. However, among the conserved region mutants, ORF67.5 m9, m11, and m12 interacted with ORF7 in the presence of ORF29 (Fig. 10A). These findings suggest that the ability of ORF67.5 to interact with ORF7 in the presence of ORF29 is necessary, but not sufficient, for the contribution of ORF67.5 to virus production. Namely, the ability of ORF67.5 to interact with ORF7 in the absence of ORF29 may be sufficient for ORF67.5 to exert the ORF67.5-mediated function in virus production. Interestingly, it has been reported that a C-terminal region of HSV-1 UL33 is important for virus production but is not required for interaction with UL28 (47). This report prompted us to ask the following question: why were the ORF67.5 conserved region mutants (ORF67.5 m9, m11, and m12) defective in the ability to complement virus production despite interacting with ORF7 in the presence of ORF29? To answer this question, we propose the following three possibilities: (i) The ORF67.5 mutants (ORF67.5 m9, m11, and m12) can form a complex with ORF7 and ORF29, but the complex cannot adopt the proper conformation to function as a terminase; (ii) ORF67.5 has an unknown function in virus production that is absent in the mutants (ORF67.5 m9, m11, and m12); and (iii) the mutants (ORF67.5 m9, m11, and m12) are unable to interact with unknown elements important for terminase function other than ORF7 and ORF29. The answer to this question is unclear and requires future clarification. Moreover, a similar question needs to be addressed for HSV-1 UL33.

We have focused our studies on the components of the KSHV terminase complex and analyzed the functions of these proteins by constructing genetically modified viruses based on reverse genetics. Our previous studies have shown that ORF7 is important for KSHV terminase function (31, 32). Moreover, characterization of ORF67.5 and its contribution to terminase function was also accomplished to some extent in this study. The remaining important issue is to elucidate the role of ORF29 in terminase function. The C-terminal exon region of ORF29 has been reported to possess DNA sequence-independent nuclease activity (48). However, the function of ORF29 in virus replication is still unknown, and this functional analysis is under way as our next research project.

## MATERIALS AND METHODS

### Plasmids.

The C-terminal 2×S-tagged ORF7 expression plasmid and the N-terminal 5×HA-tagged ORF67.5 expression plasmid have been described previously (31). The N-terminal 3×FLAG-tagged ORF67.5 expression plasmid was constructed using the insert extracted from a previously constructed N-terminal 2×S-tagged ORF67.5 expression plasmid digested with EcoRI and SalI (TaKaRa Bio, Shiga, Japan) (31). The C-terminal 3×FLAG-tagged ORF29 expression plasmid was constructed with inserts obtained by PCR using the previously constructed N-terminal 3×FLAG-tagged ORF29 expression plasmid as a template (31). All ORF67.5 mutants were constructed by PCR using an N-terminal 3×FLAG-tagged ORF67.5 expression plasmid as a template. The KOD-Plus-Neo (TOYOBO, Osaka, Japan) was used for PCR, the DNA ligation kit Mighty Mix (TaKaRa Bio) was used for ligation, and the pCI-neo mammalian expression vector (Promega, WI, USA) was used as the backbone vector. The primers used for plasmid construction are listed in Table 1. The sequences of the inserts were verified by Sanger sequencing.

**TABLE 1:** Primers for BAC mutagenesis, construction of plasmids, and qPCR

Primer name	Primer sequences (5′ → 3′)
BAC mutagenesis	
S_dORF67.5_EP	TGGCGTTCAATCTACAATAGATCGTGGGAAATAAAATTTGATGTCACGAGGCAGAAGCTGTAGGGATAACAGGGTAATCGATTT
As_dORF67.5_EP	ACATGGAGTACGCGTCTGACCAGCTTCTGCCTCGTGACATCAAATTTTATTTCCCACGATGCCAGTGTTACAACCAATTAACC
S_dORF67.5_REV_EP	TGGCGTTCAATCTACAATAGATCGTGGGAAATAAAATTTGCATGTCACGAGGCAGAAGCTGTAGGGATAACAGGGTAATCGATTT
As_dORF67.5_REV_EP	ACATGGAGTACGCGTCTGACCAGCTTCTGCCTCGTGACATGCAAATTTTATTTCCCACGATGCCAGTGTTACAACCAATTAACC
Construction plasmids	
S_EcoRI_ORF29	CATGAATTCATGCTGCTCAGCCGTCACAG
As_SalI_ORF29_C	CAAGTCGACTTGTGGGGATATGGGCTTGTAC
Fw_ORF675_m1	GACAAGCTTGGATCCGAATTCATGGAGGCCGCGGCTGCCCAGCTTCTGCCTCGTGACATG
Rv_ORF675_m1	CATGTCACGAGGCAGAAGCTGGGCAGCCGCGGCCTCCATGAATTCGGATCCAAGCTTGTC
Fw_ORF675_m2	TGGAGTACGCGTCTGACCAGGCTGCGGCTGCTGACATGCAAATTTTATTTCCCACGATCT
Rv_ORF675_m2	AGATCGTGGGAAATAAAATTTGCATGTCAGCAGCCGCAGCCTGGTCAGACGCGTACTCCA
Fw_ORF675_m3	TCTGCCTCGTGACATGCAAATTGCAGCTGCCGCGATCTATTGTAGATTGAACGCCATCAA
Rv_ORF675_m3	TTGATGGCGTTCAATCTACAATAGATCGCGGCAGCTGCAATTTGCATGTCACGAGGCAGA
Fw_ORF675_m4	ATGCAAATTTTATTTCCCACGATCTATGCTGCAGCGGCCGCCATCAACTACTGTCAGTAT
Rv_ORF675_m4	ATACTGACAGTAGTTGATGGCGGCCGCTGCAGCATAGATCGTGGGAAATAAAATTTGCAT
Fw_ORF675_m5	CGATCTATTGTAGATTGAACGCCGCCGCCGCCGCTCAGTATTTAAAAACCTTTCTCGTCC
Rv_ORF675_m5	GGACGAGAAAGGTTTTTAAATACTGAGCGGCGGCGGCGGCGTTCAATCTACAATAGATCG
Fw_ORF675_m6	AGATTGAACGCCATCAACTACTGTCAGTATGCAGCAGCCGCTCTCGTCCAGCGGGCACAG
Rv_ORF675_m6	CTGTGCCCGCTGGACGAGAGCGGCTGCTGCATACTGACAGTAGTTGATGGCGTTCAATCT
Fw_ORF675_m7	AACTACTGTCAGTATTTAAAAACCTTTCTCGTCGCGGCGGCAGCGCCTGCAGCCTGCGAT
Rv_ORF675_m7	ATCGCAGGCTGCAGGCGCTGCCGCCGCGACGAGAAAGGTTTTTAAATACTGACAGTAGTT
Fw_ORF675_m8	AGCGGGCACAGCCTGCAGCCGCCGCTGCTGCTCTGGTGTTGGAGAGCAAGGTGGACACGG
Rv_ORF675_m8	CCGTGTCCACCTTGCTCTCCAACACCAGAGCAGCAGCGGCGGCTGCAGGCTGTGCCCGCT
Fw_ORF675_m9	CCTGCAGCCTGCGATCATACTCTGGCGGCGGCGGCCAAGGTGGACACGGTAAGGCAGGTC
Rv_ORF675_m9	GACCTGCCTTACCGTGTCCACCTTGGCCGCCGCCGCCAGAGTATGATCGCAGGCTGCAGG
Fw_ORF675_m10	CCTGCGATCATACTCTGGTGTTGGAGAGCGCGGCGGCCGCGGTAAGGCAGGTCCTGCGCA
Rv_ORF675_m10	TGCGCAGGACCTGCCTTACCGCGGCCGCCGCGCTCTCCAACACCAGAGTATGATCGCAGG
Fw_ORF675_m11	TGTTGGAGAGCAAGGTGGACACGGTAGCGGCGGCCGCGCGCAAGATCGTGAGCACGGACG
Rv_ORF675_m11	CGTCCGTGCTCACGATCTTGCGCGCGGCCGCCGCTACCGTGTCCACCTTGCTCTCCAACA
Fw_ORF675_m12	GGTGGACACGGTAAGGCAGGTCCTGGCCGCGGCCGCGAGCACGGACGCCGTATTCTCCGA
Rv_ORF675_m12	TCGGAGAATACGGCGTCCGTGCTCGCGGCCGCGGCCAGGACCTGCCTTACCGTGTCCACC
Fw_ORF675_m13	GAGCACGGACGCCGTATTCTCCGAGGCGGCGGCAGCGGCCTGAGTCGACCCGGGCGGCCG
Rv_ORF675_m13	CGGCCGCCCGGGTCGACTCAGGCCGCTGCCGCCGCCTCGGAGAATACGGCGTCCGTGCTC
qPCR	
qPCR_KSHV_ORF11-Fw	TTGACAACACGCACCGCAAG
qPCR_KSHV_ORF11-Rv	AAAAATCAGCACGCTCGAGGAG
Fw_GAPDH_RTqPCR	CATCAAGAAGGTGGTGAAGCAG
Rv_GAPDH_RTqPCR	TGTCGCTGTTGAAGTCAGAGG
Fw_ORF16_RTqPCR	AGATTTCACAGCACCACCGGTA
Rv_ORF16_RTqPCR	CCCCAGTTCATGTTTCCATCGC
Fw_ORF46/47_RTqPCR	CGATCCGAATCACTGCAACG
Rv_ORF46/47_RTqPCR	CTGCTGCTTTTAGCCCGAG
Fw_K8.1_RTqPCR	TCCCACGTATCGTTCGCATTTGG
Rv_K8.1_RTqPCR	GCGTCTCTTCCTCTAGTCGTTG

### Construction of ΔORF67.5-BAC16 and Revertant-BAC16.

ΔORF67.5-BAC16 was generated from WT-BAC16 using a two-step markerless Red recombination system according to published protocols (39, 49, 50). ΔORF67.5-BAC16 contained a single cytosine deletion at position 114,586 of WT-BAC16 (GenBank: GQ994935). The primers used for this mutagenesis are shown in Table 1. The insertions and deletions of the kanamycin resistance cassette in each mutant were analyzed by EcoRV digestion and agarose gel electrophoresis. The nucleotide sequence of the region surrounding the mutated site was determined by Sanger sequencing.

### Establishment of KSHV-BAC-harboring iSLK cells.

To obtain efficient recombinant KSHV-producing cells, tetracycline (Tet)/Dox-inducible (Tet-on) RTA/ORF50-expressing iSLK cells were used as virus-producing cells (38). The iSLK cells were cultured in Dulbecco’s modified Eagle’s medium (Nacalai Tesque, Inc., Kyoto, Japan) supplemented with 10% fetal bovine serum, 1 μg/mL puromycin (InvivoGen, CA, USA), and 0.25 mg/mL G418 (Nacalai Tesque, Inc.). Thirty-six micrograms of WT-BAC16 or its mutants (ΔORF67.5-BAC16 and Revertant-BAC16) were transfected into iSLK cells (1 × 10^6^ cells) by the calcium phosphate method. The transfected cells were selected under 1 mg/mL hygromycin B (Wako, Osaka, Japan) to establish Dox-inducible recombinant KSHV-producing cell lines (WT-iSLK, ΔORF67.5-iSLK, and Revertant-iSLK).

### Characterization of mutant KSHVs.

Each measurement was performed using previously described methods (32). Briefly, iSLK cells harboring WT or each KSHV mutant were treated with 8 μg/mL Dox and 1.5 mM SB for 72 h to induce lytic replication.

For measurement of viral gene expression, lytic-induced or uninduced cells (5 × 10^5^ cells in a 6-well plate) were harvested with 500 μL of RNAiso Plus (TaKaRa Bio). Total RNA was extracted, and cDNA was synthesized from 160 ng of total RNA using ReverTra Ace qPCR RT master mix (TOYOBO). RT-qPCR was performed using the synthesized cDNAs as the template.

For quantification of intracellular viral DNA replication, iSLK cells (3.5 × 10^4^ cells in a 48-well plate) were induced or not induced and harvested. Viral genomic DNA was purified from the harvested cells using a QIAamp DNA blood minikit (Qiagen, CA, USA). The viral genome copy number was quantified by qPCR and normalized to the amount of total DNA.

For quantification of extracellular encapsidated viral DNA, iSLK cells (1.5 × 10^5^ cells in a 12-well plate) were induced, and the culture supernatants were harvested and centrifuged to remove debris. The supernatants were treated with DNase I (New England Biolabs, MA, USA), and encapsidated viral DNA was extracted from the supernatants using a QIAamp DNA blood minikit (Qiagen). The purified KSHV genomic DNA was quantified by qPCR.

For measurement of infectious virus, a supernatant transfer assay was performed. iSLK cells (1 × 10^6^ cells on a 6-cm dish) were induced, and the culture supernatants and cells were collected. The supernatants and cells were centrifuged, and the supernatant was mixed with trypsinized HEK293T cells (7.5 × 10^5^ cells). Polybrene (8 μg/mL; Sigma-Aldrich, MO, USA) was added to the cell mixtures, which were then placed into 6-well plates. The plates were centrifuged at 1,200 × *g* for 2 h at room temperature and incubated for 24 h at 37°C. GFP-positive cells were analyzed with a flow cytometer (FACSCalibur, Beckton Dickinson, CA, USA) using CellQuest Pro software (Beckton Dickinson).

### qPCR.

All qPCR assays were performed using THUNDERBIRD Next SYBR qPCR mix (TOYOBO). To measure KSHV genome copy numbers, qPCR was performed using the KSHV-encoded ORF11-specific primers shown in Table 1. Table 1 also lists the primers used to measure viral gene expression. Relative mRNA expression levels were determined by the delta-delta threshold cycle (ΔΔ*C_T_*) method and were normalized to *GAPDH* mRNA levels.

### Complementation assay.

The complementation assay was performed as previously described (32). Briefly, iSLK cells were transfected with each plasmid using ScreenFect A-plus (Wako) and simultaneously cultured with medium containing 8 μg/mL Dox and 1.5 mM SB. After 72 h, virus production and infectivity were assessed according to the methods described in the section Characterization of mutant KSHVs.

### Western blotting and antibodies.

Western blotting was performed as previously described (51). Briefly, cells were washed with phosphate-buffered saline (PBS), lysed with SDS sample buffer (containing 2% [vol/vol] 2-mercaptoethanol), sonicated for 10 s, reduced at 60°C for 10 min, and subjected to SDS-PAGE and Western blotting. When lytic-induced cells were used, the cells were treated with 8 μg/mL Dox and 1.5 mM SB for 72 h. Anti-KSHV ORF67.5 rabbit pAb was generated by GL Biochem, Shanghai, China, using the synthetic peptide Cys-KIVSTDAVFSEARAR (ORF67.5: aa 65 to 79) as the antigen. Anti-KSHV ORF67.5 rabbit pAb was purified from the immunized rabbit serum using antigen peptide affinity chromatography. The following primary antibodies were used: anti-K-bZIP mouse monoclonal antibody (MAb; F33P1; Santa Cruz Biotechnology, TX, USA), anti-K8.1 A/B mouse MAb (4A4; Santa Cruz Biotechnology), anti-ORF21 rabbit pAb (previously produced in our laboratory) (52), anti-β-actin mouse MAb (AC-15; Santa Cruz Biotechnology), anti-FLAG mouse MAb (FLA-1; MBL, Nagoya, Japan), anti-S rabbit pAb (sc-802; Santa Cruz Biotechnology), and anti-HA mouse MAb (TANA2; MBL). Anti-mouse IgG-horseradish peroxidase (HRP; NA931; Cytiva, Tokyo, Japan) and anti-rabbit IgG-HRP (7074; Cell Signaling Technology, MA, USA) were used as the secondary antibodies.

### Pulldown assay.

HEK293T cells (2 × 10^6^ cells on a 10-cm dish) were transfected with 12 μg of plasmid DNA and 36 μg of polyethylenimine hydrochloride/PEI MAX (Polysciences, Inc., PA, USA). The cells were then cultured for 20 h. Next, the cells were lysed with 1.5 mL of lysis buffer (50 mM Tris-HCl [pH 8.0], 120 mM NaCl, glycerol [1% vol/vol], Nonidet P-40 [0.2% vol/vol], and 1 mM dithiothreitol). The cell extracts were incubated with 20 μL of S-protein-immobilized agarose beads for 1 h, and the beads were washed three times with lysis buffer. The washed beads were resuspended in 15 μL of SDS-PAGE sample buffer containing 2-mercaptoethanol (2% [vol/vol]). The precipitates were detected by Western blotting. The band intensities of the detected proteins were measured using ImageJ software (NIH, Bethesda, MD, USA).

### Electron microscopy.

To observe intracellular capsid formation, iSLK cells were cultured for 48 h in medium containing 8 μg/mL Dox and 1.5 mM SB. The cultured cells induced for virus production were washed with PBS and trypsinized for cell detachment. The detached cells were resuspended in medium, washed with PBS, and pelleted. The samples were then fixed in 2% glutaraldehyde in PBS (pH 7.2) for 2 h on ice, followed by incubation in 1% osmium tetroxide in PBS at 4°C for 1.5 h. The fixed samples were washed five times in PBS and dehydrated in graded ethanol. The samples were then embedded in an epoxy resin (Nisshin EM Co., Ltd., Tokyo, Japan) and processed by routine electron microscopy. Ultrathin sections were prepared using a Porter Blum ultramicrotome (Reichert-Nissei ULTRACUT-N; Leica, Wetzlar, Germany) and mounted on a copper grid (200 mesh) supported by a carbon-coated collodion film. The ultrathin sections were double stained with uranyl acetate (4% [wt/vol]) and lead citrate for 10 min and 3 min, respectively. After each staining procedure, the sections were washed three times in distilled water. All sections were observed under a Hitachi H-7800 transmission electron microscope.

### Southern blotting.

Southern blotting was performed as previously described (32, 37). Briefly, iSLK cells (2 × 10^6^ cells in a 10-cm dish) were treated with 8 μg/mL Dox and 1.5 mM SB for 72 h. Next, the cells were washed with PBS and suspended in TNE buffer (10 mM Tris-HCl [pH 7.5], 100 mM NaCl, and 1 mM EDTA), followed by an overnight incubation at 60°C with SDS (0.3% [wt/vol]) and proteinase K (100 μg/mL). The cell extracts were then incubated with RNase (100 μg/mL) at room temperature for 30 min. DNA was isolated from the cell extracts by phenol-chloroform extraction and ethanol precipitation. The pellets were then resuspended in 1 × Tris-EDTA (TE) buffer. The DNA samples (10 μg each) were digested with EcoRI (TaKaRa Bio) and SalI (TOYOBO) overnight, followed by electrophoresis on a 0.7% agarose 1 × Tris-borate-EDTA (TBE) gel. The gel was denatured in alkaline denaturing buffer (0.5 N NaOH and 1.5 M NaCl) for 45 min and transferred to a Hybond-N+ membrane (Cytiva) by capillary action overnight in the presence of alkaline transfer buffer (0.4 N NaOH and 1 M NaCl). The transferred DNA was cross-linked, and the membrane was treated with a DIG-High Prime DNA labeling and detection starter kit II (Roche, Basel, Switzerland) according to the manufacturer’s protocol. The membrane was hybridized overnight at 42°C in DIG Easy Hyb buffer with a digoxigenin (DIG)-labeled TR probe. Next, the membrane was washed twice with 2 × saline sodium citrate buffer (SSC) containing 0.1% SDS at room temperature followed by two additional washes with 0.5 × SSC containing 0.1% SDS at 68°C. The membrane was blocked, incubated with anti-DIG antibody, and detected by CSPD. The blot was then exposed to X-ray film (Fuji film, Tokyo, Japan).

### Protein structure prediction.

The structure of the monomeric HSV-1 terminase complex (Protein Data Bank [PDB]: 6M5R) (21), determined by cryo-EM, was visualized with the molecular visualization open-source software program PyMOL (version 2.5.0). The structure of the monomeric KSHV terminase complex, determined by the deep learning algorithm AlphaFold 2.2.0 (https://github.com/deepmind/alphafold) in the local environment (53), was also visualized with PyMOL.

### Statistics.

The statistical significance was determined by one-way analysis of variance (ANOVA) followed by a Dunnett’s test and was evaluated using GraphPad Prism 7 software (GraphPad Software, CA, USA).

### Data availability.

Underlying data and the accession numbers are available in the main text. All other raw data will be shared upon request.
